# Growth and Progression of TRAMP Prostate Tumors in Relationship to Diet and Obesity

**DOI:** 10.1155/2012/543970

**Published:** 2012-12-04

**Authors:** Melissa J. L. Bonorden, Michael E. Grossmann, Sarah A. Ewing, Olga P. Rogozina, Amitabha Ray, Katai J. Nkhata, D. Joshua Liao, Joseph P. Grande, Margot P. Cleary

**Affiliations:** ^1^The Hormel Institute, University of Minnesota, Austin, MN 55912, USA; ^2^Department of Pathology and Laboratory Medicine, Mayo Clinic, Rochester, MN 55905, USA

## Abstract

To clarify effects of diet and body weight on prostate cancer development, three studies were undertaken using the TRAMP mouse model of this disease. In the first experiment, obesity was induced by injection of gold thioglucose (GTG). Age of prostate tumor detection (*~*33 wk) and death (*~*43 wk) was not significantly different among the groups. In the second study, TRAMP-C2 cells were injected into syngeneic C57BL6 mice and tumor progression was evaluated in mice fed either high-fat or low-fat diets. The high fat fed mice had larger tumors than did the low-fat fed mice. In the third study, tumor development was followed in TRAMP mice fed a high fat diet from 6 weeks of age. There were no significant effects of body weight status or diet on tumor development among the groups. When the tumors were examined for the neuroendocrine marker synaptophysin, there was no correlation with either body weight or diet. However, there was a significant correlation of the expression of synaptophysin with earlier age to tumor detection and death. In summary, TRAMP-C2 cells grew faster when the mice were fed a high-fat diet. Further synaptophysin may be a marker of poor prognosis independent of weight and diet.

## 1. Introduction

Epidemiological studies suggest that diet and increased body weight play a role in the development of prostate cancer [1]. Also, intake of high-fat dairy products and red meat appear to be linked to an increased risk of prostate cancer [2]. However, men with this pattern of eating also tend to consume fewer fruits and vegetables. Thus, while it seems clear that a diet high in fruits and vegetables and low in high-fat dairy and meat is linked to a decreased risk of prostate cancer [1], it has been hard to clarify the exact role of fat in prostate cancer progression. With respect to body weight, Grönberg et al. reported in a prospective study of Swedish men that there was a positive trend for both increased body mass index (BMI) and food consumption to be associated with a higher risk of prostate cancer [3]. Other studies have supported that higher BMI and/or weight gain increase the risk of prostate cancer [4–10]. Although, not all studies have found elevated BMI to increase the risk of prostate cancer, Bergström et al. concluded that the obtained relative risk values corresponded to a 6% increase in risk of prostate cancer for an overweight man compared with a normal weight and to a 12% increase in risk for an obese man when looking at all studies (North America and European) and that the proportion of prostate cancers attributable to overweight and obesity among European men is 4% [11]. Obesity was reported to be associated with higher prostate cancer grade at diagnosis [12, 13], increased tumor volume at radical prostatectomy [14, 15] as well as with higher recurrence rates [16]. Abdominal adiposity, waist circumference and BMI were also associated with greater prostate volume as well as younger age at diagnosis [17]. In addition, mortality from prostate cancer has been reported to be increased with elevated body weights [18]; however, other studies have not found this [17]. In an examination of a recent meta-analysis of prostate cancer as it related to obesity, it was found that a 5 kg/m^2^ increase in BMI was associated with a 15–20% higher risk of dying of prostate cancer depending on what type of study was conducted and a 21% increased chance of biochemical relapse [19]. In addition, a second meta-analysis determined that obesity appears to result in an increased risk of advanced prostate cancer [20]. The potential role of body fat and body weight in the development and progression of prostate cancer is of interest given that the incidence of overweight/obesity is increasing throughout the world.

Assessment of the role of diet and body weight in prostate cancer, a disease which takes decades to develop, is a difficult undertaking. Most frequently recent dietary intakes and body weight are obtained at or near the time of diagnosis. However, we hypothesize that data obtained at this point may have little relevance to the impact that diet and body weight have on the initiation and then long-term progression of the disease process which extends over many decades. Also, when prostate cancer is diagnosed it can be at various stages and have multiple etiologies. Overall these factors can result in the inability to identify diet and lifestyle components that might preferentially affect subsets of prostate cancer and necessitate the utilization of large numbers of subjects to obtain valid results. Prospective studies try to address some of these issues but again large numbers of subjects must be followed and these studies are very expensive to maintain.

To more clearly understand how diet and body weight impact prostate cancer, it is necessary to relate these factors with different types of prostate cancer. Neuroendocrine (NE) differentiation has been detected in a subset of prostatic adenocarcinomas [21]. NE positive cells play important regulatory roles and increase proliferation in nearby cells due to growth factor secretion [22]. Prostate cancer can differentiate from non-NE to NE during cancer progression. NE differentiation has been associated with more aggressive tumors that have a greater potential for hormone resistance [23] and as such NE status is currently being evaluated for its possible prognostic value [24, 25]. Current work has shown that patients with Gleason score 8–10 prostate cancer that have >1% NE positive diseases have inferior clinical outcomes when they are treated with radiotherapy due to an increase in distant disease failure [26]. The number of NE positive cells was correlated with the Gleason score and high numbers of NE positive cells were associated with early treatment failure [27]. As noted above obesity has been associated with increased risk of more aggressive prostate cancer, higher rates of biochemical relapse, and prostate cancer-specific death. It has also been reported that consumption of methylseleninic acid by TRAMP mice resulted in a decrease in the number of NE positive tumors [28]. This suggested to us that diet and body weight might also play a role in the NE status of the TRAMP tumors.

**Figure 1:**
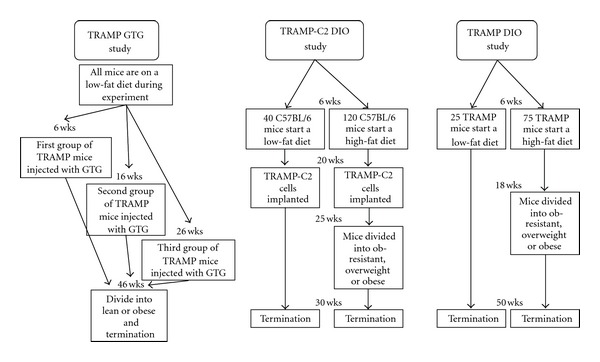
Flow chart of the three different studies whose results are discussed in this paper. Boxes at top show name of study. Arrows show movement of groups over time. Wks is weeks of age. Termination was either the wks stated or when the mice reached experimental endpoints related to tumor size and health.

Introduction of the TRAMP (transgenic adenocarcinoma mouse prostate) mouse on the C57BL6 background provides an animal model that shares many characteristics with human prostate cancer including the fact that a subset of the tumors are NE marker positive [29–31]. We proposed that this model would be useful to assess the effect of obesity on prostate cancer development because of these similarities. To assess the effects of diet and obesity on prostate cancer, three different experiments were undertaken. In the first study, goldthioglucose (GTG) was administered to TRAMP mice to induce obesity at specific ages to determine the effect of obesity on cancer development and progression over different periods of time. This is called the TRAMP GTG study. The second and third studies utilized a diet-induced obesity (DIO) regimen similar to that which was previously used to study mammary tumor development [32–35]. In both our laboratory and others, it has been reported that C57BL6 mice fed a high-fat diet gain weight and become overweight or obese, but a subset will be obesity-resistant despite the fact that our previous studies did not find a significant difference in caloric intakes between the groups [32]. The obesity-resistant mice and the low-fat fed mice will have similar weights [33, 36]. This protocol provides the opportunity to compare mice that have different body weights even though they are consuming the same diet. In addition, we also included a group of mice fed a low-fat diet whose weights were similar to the obesity-resistant high-fat fed mice. This allowed us to study the effect of diet on prostate cancer in the absence of differences in weight. In the second study TRAMP-C2 tumor cells were injected into the flanks of C57BL6 male mice and tumor growth was monitored [37, 38]. This study is called the TRAMP-C2 DIO study. In the third study, TRAMP mice were fed the diets and the autochthonous tumors directly followed. This is called the TRAMP DIO study.  Figure 1 shows an overview of the three studies. The results of the three studies were evaluated to gain a better understanding of the roles of diet and body weight in prostate cancer growth and progression.

## 2. Methods and Materials

### 2.1. General Mouse Procedures

Breeding pairs of TRAMP mice were obtained from Jackson Laboratory (Bar Harbor, ME). Nontransgenic male C57BL6 mice were mated with heterozygous C57BL6 (TRAMP +/−) females which produced both transgenic and nontransgenic offspring. Pups were maintained with their mothers until 3 week of age and then removed and housed with same sex littermates prior to genotyping. Mice were genotyped to identify the presence of the PB-SV40 T antigen transgene (0.6 kb) using the oligonucleotide primer PB1-forward (5′ CCG GTC GAC CGG AAG CTT CCA CAA GTG CAT TTA 3′) and the complementary primer TAG-reverse (5′ CTC CTT TCA AGA CCT AGA AGG TCC A 3′) as previously described [39]. From weaning, mice were fed AIN-93 M diet. Mice were followed for prostate cancer detection and were euthanized due to tumor formation or when they reached designated ages. These studies were approved by the University of Minnesota IACUC and the Hormel Institute Animal Facility is AAALAC accredited.

### 2.2. TRAMP GTG Study

Reports in the literature indicated doses of 0.5–2.0 g/kg body weight of GTG produced obesity in a portion of treated mice independent of age and sex [40–46]. We conducted a pilot study using wild-type C57BL6 mice and based on the results 0.8 g/kg of GTG was selected for further use. Mice were assigned to be injected with GTG at either 6, 16, or 26 weeks of age. Due to unforeseen problems with mortality associated with the 0.8 g/kg dose in the TRAMP mice, the dose was lowered to 0.5 g/kg. Following injection the mice were fasted for 24 hours and then given glucose supplemented water for one week. The designated end point of this study was 48  weeks of age.

### 2.3. TRAMP-C2 DIO Study

We obtained C57BL6 wild-type male mice (*n* = 160) from Jackson Laboratory, Bar Harbor ME. Mice (*n* = 40) were either maintained on a low-fat diet (AIN-93 M) [35, 47] or from six weeks of age (*n* = 120) were fed AIN-93 M-high-fat diet [35]. At 20  weeks of age mice were implanted with the TRAMP-C2 cells (3 × 10^6^) in the left flank. Mice were weighed and palpated for tumors over the next 10 weeks. At the termination of the study the tumors were removed, measured, and weighed and then processed for histopathology. Epididymal and retroperitoneal fat pads were removed as well as genitourinary tracts (GUT) and prostates. Two of the high-fat fed mice were removed from the study due to nonexperimental related illnesses. All of the low-fat fed mice finished the study. Four additional mice were removed from the high-fat fed group analysis due to no discernable tumor mass.

### 2.4. TRAMP DIO Study

TRAMP mice were either maintained on AIN-93 M throughout the study or were switched to the high fat diet at 6 weeks of age. Based on body weight gain from 6–18 week of age, high fat diet fed mice were divided into three groups designated as obesity-prone, the heaviest third, overweight, the middle third, and obesity-Resistant, the lightest third. This designation was based on earlier studies in female mice in mammary tumor studies [32, 33]. An earlier time point for initiating the high-fat diet and for determining group status was used due to the earlier onset and more aggressive nature of this malignancy as compared to the mammary tumor model.

### 2.5. Study Terminations

Mice were euthanized at designated ages in each of the protocols or when disease burden dictated as assessed by weight loss, palpation of large abdominal masses, blood in the urine or unkempt/scruffy appearance. Mice were anesthetized with isoflurane and the liver, heart, lymph nodes, lungs, kidneys, spleen, GUT, epididymal, and retroperitoneal fat pads were removed and weighed. Visible tumors from the GUT were removed and weighed. Carcass weight was calculated by subtracting organ and tumor weight from final body weight. Samples from organs and tumors were placed in 10% neutral buffered formalin before being embedded in paraffin. Slides were prepared from tissue sections at a thickness of 5 *μ*m, deparaffinized in xylene, rehydrated in a graded series of ethanol solutions, and rinsed in tap water. Following H & E staining, histopathological analysis was conducted on sections in a blinded fashion.

### 2.6. Western Blot Analysis

Whole-cell extracts were obtained as per the manufacturer's instructions for Novagen Phosphosafe (Merck KGaA, Darmstadt, Germany) extraction reagent. Antibodies were obtained from Cell Signaling Technology, Inc. (Danvers, MA). Blots were treated as previously described [48] and then visualized with a STORM 840. 

### 2.7. Statistical Analyses

All data analyses were performed using GraphPad Prism version 4.0. Either *t*-tests or one-way analysis of variance was utilized for the studies with significant differences defined as at least *P* < 0.05. If the one-way analysis of variance was significant then the Newman-Keuls posttest was used to compare all groups. Significance between specific groups using Newman-Keuls was defined as at least *P* < 0.05.

## 3. Results

### 3.1. TRAMP GTG Study

Our initial goal was to determine how the age of onset of obesity would influence prostate tumor growth or progression. To do this the male TRAMP mice were injected with GTG at various ages. A preliminary study in wild-type C57BL6 mice indicated that a GTG dose of 0.8 mg/kg resulted in 67% (8 of 12) survival with 62.5% (5 of 8) of the surviving mice becoming obese. However when 26-week-old TRAMP mice were injected with this GTG dose the survival rate was only 42% (14 of 33). Of the 14 surviving mice 8 were classified as obese and 6 lean, based on body weight gain relative to saline injected control mice. For the 16 and 6 week cohorts we lowered the dose of GTG to 0.5 g/kg for some of the mice in an attempt to improve survival rates. However, in the 16 week cohort survival, rates were only 13% and 9% for 0.8 and 0.5 g/kg doses, respectively. Of the three total survivors only one became obese. Therefore, this group was not useful for statistical analysis and will not be further discussed. When 6 week old TRAMP mice were injected with 0.8 g/kg body weight of GTG no mice survived however of those injected with the lower dose of 0.5 g/kg 23% survived (7 of 31). Of the 7 surviving mice injected at 6 weeks of age 4 were obese and 3 were lean.

Body weights of the GTG-obese mice were substantially heavier than those of either GTG-lean or PBS-control mice and body weights of the GTG-obese mice were similar regardless of the age of GTG injection (Table 1). Fat pad weights followed a similar pattern with those of the GTG-obese mice being two times heavier then those of the GTG-lean and PBS-control mice (data not shown). In contrast to body and fat pad weights, GUT weights were not significantly different among the groups regardless of the age of GTG injection. Further, within each GTG cohort, there was no effect of body weight on the age of prostate tumor detection or age of the mice at death. Mice injected with GTG at 26 weeks of age tended to be a little older at both these time points than the mice injected at younger ages (Table 1). This is probably due to survival advantage of those mice having survived until 26 weeks of age in order to be enrolled in the study. 

Pathology results for GUT and metastases rates for the mice injected with GTG at six weeks of age indicated a trend for the GTG-lean mice to have a higher rate of poorly differentiated tumors compared to the GTG-obese and PBS-controls (Table 1). The latter two groups tended to have more well-differentiated tumors. The metastases rate tended to be lowest for the GTG-obese mice compared to the other two groups. For the 26-week cohort the majority of the tumors in all three groups were either well or moderately defined and the metastases rate was lowest for the GTG-obese mice. Due to the small numbers of mice, it was not possible to do statistical calculations of the pathology results. Interestingly, overall the obese mice tended to have reduced metastases rates compared to the two lean groups, 17% versus 38% and 41%.

### 3.2. TRAMP-C2 Cell DIO Study

**Figure 2:**
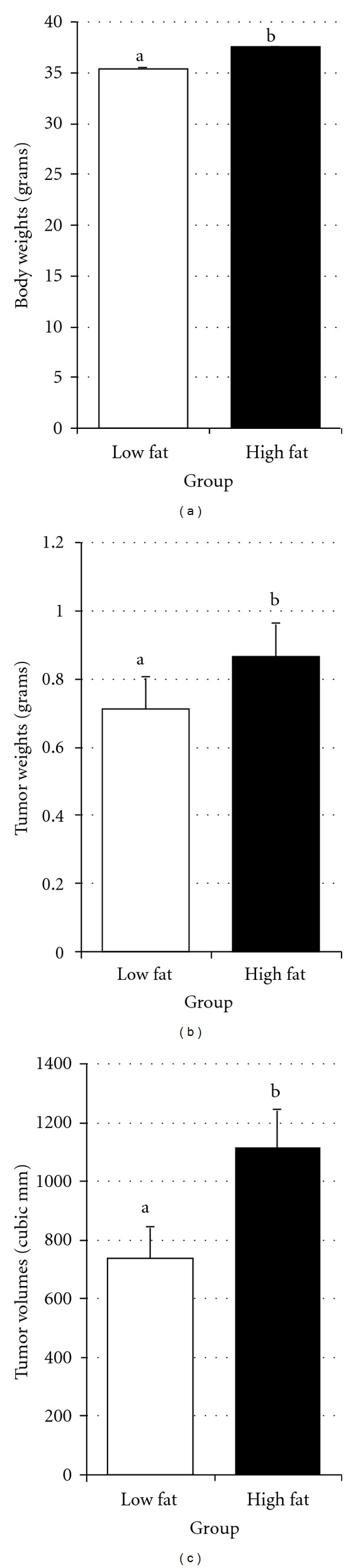
Comparison of TRAMP C-2 tumor cell growth in low-fat fed (open bars *N* = 40) and high-fat fed (filled bars *N* = 134) mice. (a) Body weights at 25 weeks of age with animal weights in grams along the *y*-axis (*t*-test *P* < 0.003). (b) Tumor weights at termination with weights in grams shown along the *y*-axis (*t*-test *P* < 0.01). (c) Tumor volume at termination with size in mm cubed shown along the *y*-axis (*t*-test *P* < 0.0007). Bars represent standard error and letters show significant differences.

The TRAMP-C2 prostate cancer cell line was derived from the tumor of a TRAMP mouse [37]. These cells form tumors in syngeneic male C57BL6 mice.  Figure 2(a) shows the weights of the low-fat (open bar) and high-fat (filled bar) groups of mice at 25 weeks of age (this age was used to avoid potential weight loss due to tumor growth). The mice in the low-fat group averaged 35.4 grams and the mice in the high-fat group were significantly heavier at 37.5 grams (*P* < 0.003). Tumor weights are presented in Figure 2(b). It can be seen that the high-fat fed mice had heavier tumors (0.867 g) than the low-fat fed mice (0.710 g) (*P* < 0.01) and as shown in Figure 2(c) the average tumor volume of the high-fat fed mice was higher (1115.7 mm^3^) as compared to the low-fat fed (738.6 mm^3^) mice (*P* < 0.0007).

**Figure 3:**
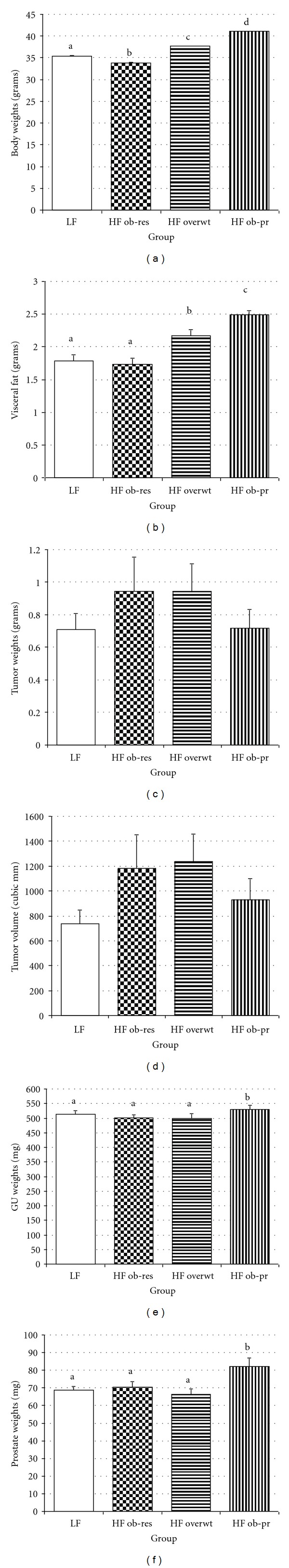
TRAMP-C2 tumor growth based on body weight and diet. Body weights of low-fat fed (open bars *N* = 40), high-fat fed obesity-resistant (checkered bars *N* = 38), high-fat fed overweight (horizontal bars *N* = 38), and high-fat fed obesity-prone (vertical bars *N* = 38) at 25 weeks of age. (a) Average body weights with grams along the *y*-axis (overall ANOVA *P* < 0.0001). (b) Average fat pad weights at termination in grams *y*-axis (overall ANOVA *P* < 0.0001). (c) Tumor weights at termination in grams (*y*-axis and (d)). Tumor volume at termination in mm cubed (*y*-axis). (e) Genitourinary tract (GU) weights with milligrams along the *y*-axis (*P* < 0.01). (f) Prostate weights with milligrams along the *y*-axis (*P* < 0.01). Bars represent standard error and letters show significance differences.

Next we divided the mice fed the high-fat diet into three body weight categories, obesity-resistant, overweight, and obesity-prone based on their weights at week 25 with 1/3 of the mice placed into each group as we have done previously [33]. The ANOVA for the mouse weights was *P* < 0.0001. All groups were significantly different from each other (*P* < 0.001). The low-fat fed mice weighed on average 35.4 grams (Figure 3(a)), the high-fat fed obesity-resistant mice averaged 33.8 grams which was actually significantly lower compared to the low-fat fed mice. The overweight mice averaged 37.6 grams and the obesity-prone mice weighed an average of 41.1 grams. Similar results were obtained for the visceral fat pads (Figure 3(b)). The ANOVA for the visceral fat pads was *P* < 0.0001. The lightest visceral fat pads were from the low-fat fed mice (1.78 grams) and the high-fat fed obesity-resistant mice (1.73 grams) and these were not significantly different from each other. The fat pad weights of the overweight and obesity-prone mice averaged 2.16 grams and 2.49 grams respectively and were significantly different from each other as well as from the low-fat fed and high-fat fed obesity-resistant mice.

We examined the TRAMP-C2 tumors harvested from the mice and compared the weights and volumes relative to body weight groups.  Figure 3(c) shows that the TRAMP-C2 tumors from the low-fat fed and obesity-prone mice were the lightest. The tumors from the obesity-resistant and the overweight mice were heavier. However, the differences in tumor weight were not significant between any specific groups. When the tumor volumes were computed the low-fat fed mice had the smallest tumors followed by the obesity-prone mice with the obesity-resistant and overweight mice having the largest tumors by volume (Figure 3(d)). Again, none of the tumor volumes were significantly different between any groups.

Because the TRAMP-C2 cells were injected subcutaneously, we were able to examine the effects of body weight and a high-fat diet on normal GUT and prostates. We found that GUT weights from the high-fat fed obesity-prone mice were significantly heavier (*P* < 0.01) than the GUT weights from any of the other groups (Figure 3(e)). We also found that the high-fat fed obesity-prone mice had significantly heavier prostates (*P* < 0.01) as compared to any of the other groups (Figure 3(f)).

### 3.3. TRAMP DIO Study

**Figure 4:**
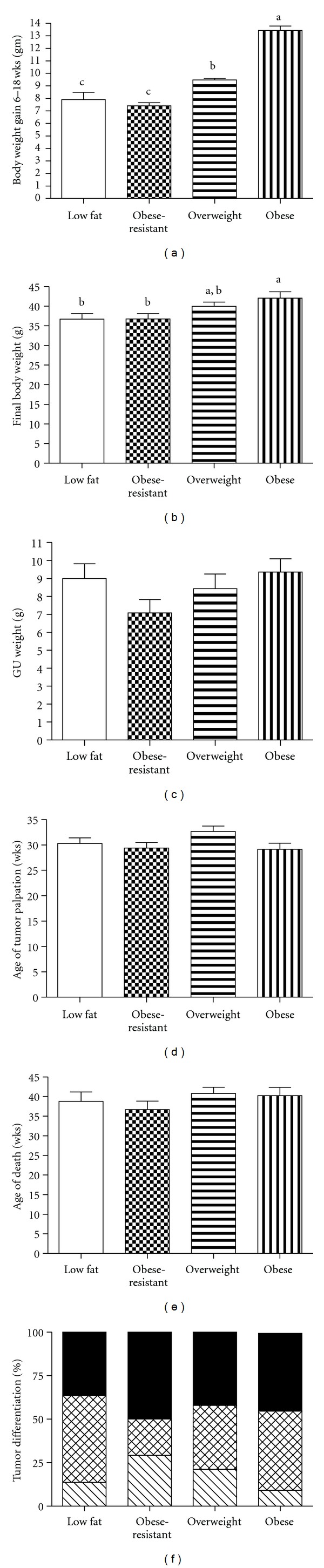
Autochthonous tumor growth in mice with diet-induced obesity. Mice were fed a low-fat diet (open bars) or a high-fat diet and divided into three groups designated as obesity-resistant (checkered bars), the lightest third (*N* = 24). Overweight (horizontal bars), the middle third (*N* = 24) and obese (vertical bars) the heaviest third (*N* = 24). (a) Weight gain from 6–18 weeks (overall ANOVA *P* < 0.0001) with weight in grams shown along the *y*-axis. (b) Final body weight with grams along the *y*-axis (overall ANOVA *P* = 0.0158). (c) GUT weight with grams along the *y*-axis (overall ANOVA *P* < 0.1723). (d) Age of tumor palpation with weeks along the *y*-axis (overall ANOVA *P* < 0.1235). (e) Age at death with weeks along the *y*-axis (overall ANOVA *P* < 5196) and (f) GUT pathology with PIN/well differentiated (angled lines in bar), moderately differentiated (hatched lines in bar), and poorly differentiated (filled part of bar). Each bar represents one group with the percent of different tumor grades shown along the *y*-axis. Error bars are standard error and letters show significance differences.

To investigate the effect of diet and body weight directly on prostate tumors, we fed TRAMP mice low or high-fat diets and examined the growth of their autochthonous tumors. At euthanasia the body weights of the high-fat fed mice were not statistically different from the low-fat mice (data not shown). Therefore, based on body weight gain from 6–18 weeks of age, TRAMP mice fed the high-fat diet were divided into three groups. The obesity-prone mice gained significantly more weight than the other groups of mice (Figure 4(a)). The overweight mice gained an intermediate amount of weight that was significantly different from the obesity-prone and the obesity-resistant groups while the obesity-resistant mice gained an amount of weight similar to that of the low-fat fed mice. The fat pads weights were heaviest in the mice designated as obesity-prone while the overweight mice were intermediate and the obesity-resistant mice had fat pad weights that were not significantly different than those of the low-fat fed mice (Figure 4(b)). There were no significant effects of GUT weight relative to body weight among the groups (Figure 4(c)). Additionally, there was no effect of body weight or diet on either age to tumor detection (Figure 4(d)) or age at death (Figure 4(e)). Pathological analysis of GUT or dissected tumors revealed that when the highest grade tumors (poorly differentiated > moderately differentiated > well differentiated > PIN) were counted low-fat and obesity-prone mice had 13.6% and 9.1% lesions graded as either PIN or well-differentiated adenocarcinoma, respectively. The low-fat fed mice also had 50% moderately differentiated tumors and 36.4% poorly differentiated tumors while the obesity-prone mice had 45.5% moderately differentiated and 45.5% poorly differentiated tumors. The combined incidence of moderately or poorly differentiated tumors was 70.8% for obesity-resistant mice and 91% for obesity prone mice while overweight mice had 78.9% (Figure 4(f)). These data are summarized in Table 2.

To better understand the relationship of obesity to more aggressive prostate tumors, we examined the neuroendocrine (NE) status of the autochthonous tumors. We evaluated the same groups as in Table 2 as well as a group that was an aggregate of all mice fed the high-fat diet regardless of weight (All High-Fat fed). We found that there were no significant differences in NE status between the groups regardless of body weight or diet consumed (Table 3). However, when we compared the characteristics of the NE positive tumors verses NE negative tumors within each group significant differences were found. The initial body weights and amount of weight gained were similar within groups regardless of NE status. However, mice positive for NE were significantly younger at tumor detection than mice that were NE negative. This was true for all groups except for the obesity-resistant group where it was only a trend (Table 3). In addition, the age of death was significantly younger within all of the groups if the tumors were NE positive except for the Obesity-Resistant group where it was again only a trend. Interestingly, the final body weights were lower in the NE positive animals. This is likely due to the fact that these mice were younger at euthanasia as body weight of the mice correlated with their age throughout the course of the study. The GUT weights were significantly lower in the NE positive mice in the Obesity-Prone, All high-fat fed and low-fat fed groups (Table 3). The same trend was seen in the overweight and obesity-resistant groups but did not reach significance (Table 3). When NE status was evaluated with respect to tumor grade, 100% of the NE positive tumors were poorly differentiated. In contrast, NE negative tumors were multiple grades with significantly fewer poorly differentiated tumors in the obesity-prone, high-fat and low-fat groups compared to NE positive tumors. There was a trend to fewer poorly differentiated tumors when comparing the NE negative tumors to the NE positive tumors from the obesity-prone and obesity-resistant groups that did not reach statistical significance.

## 4. Discussion

We utilized three different protocols to examine questions relating to body weight and prostate cancer development or progression which have led to a number of conclusions (Table 4). In the first study our goal was to induce obesity at three different ages to investigate the effect of body weight changes at different points in development and progression of autochthonous tumors in TRAMP mice. GTG was chosen to induce obesity which was independent of genetic or dietary influences. Despite preliminary studies using wild type C57BL6 mice to determine a dosing regimen for the GTG with low mortality and effective obesity rates, administering this compound to TRAMP mice resulted in high mortality providing limited numbers of experimental mice to follow. However, a few interesting observations were made for the mice that did survive. For example, the GTG-obese mice injected at 6 weeks of age had a delay in tumor detection compared to the GTG-lean mice and a delayed age at death although GUT weight was not impacted by body weight. Tumor differentiation was improved and metastases rate was reduced in GTG-obese mice compared to the PBS-control mice. For the mice injected at 26 weeks of age, age of tumor detection, age at death, and GUT weights were similar in all three groups. There was, however, a trend for the GTG-obese mice to have an improved tumor differentiation profile compared to both lean groups and to have a reduced metastasis rate compared to the control saline injected mice. This was an unexpected result; however, it may be explained by the fact that this was a trend and as such not statistically significant. Alternatively, it is possible that because the promoter that induces the TRAMP oncogene is testosterone driven these mice require a specific period of time to progress from well differentiated to poorly differentiated tumors and that the obese mice have lower levels of testosterone such as has been found in obese human males [49, 50] thereby reducing the number of poorly differentiated tumors in the animals.

In the second and third studies, a diet-induced obesity (DIO) protocol which we had used to study mammary tumor development in transgenic mice as well as in a xenograft study was followed [32–35]. Previous studies have shown that although most C57BL6 mice fed a high-fat diet will gain weight and become overweight or obese, some will stay in the body weight range of low-fat fed mice [32, 36]. This provides the opportunity to compare mice of the same body weight consuming diets of different composition as well as to compare mice fed the same diet but with different body weights. In the second study we utilized syngeneic TRAMP-C2 cells to form subcutaneous tumors as has been done previously [37, 38]. Consumption of a high fat-diet by male mice resulted in TRAMP-C2 tumors that weighed significantly more (Figure 3(c)) and had a significantly larger volume (Figure 3(d)) as compared to the tumors from mice fed a low-fat diet. When we examined the effects of body weight status of mice on tumor size obesity-prone mice and low-fat fed mice were not significantly different. The weights and sizes of the tumors from the overweight and obesity-resistant mice were not statistically different from each other but tended to be higher than those of the low-fat and obesity-resistant-prone mice (Figures 3(c) and 3(d)) suggesting a complex interplay between body weight and diet. It is possible that the significant differences seen in tumor size and volume between high-fat fed mice and low-fat fed mice are due to diet. This hypothesis is supported by the facts that obesity-prone mice did not have a change in tumor size or volume compared to low-fat fed mice suggesting that the obesity-prone mice may have some protective effect generated by increased levels of body fat against the high-fat diet. On the other hand the high-fat fed obesity-resistant and overweight animals while more similar in weight to the low-fat fed mice had higher tumor weights and volumes than the low-fat diet animals suggesting that the high-fat diet is tumor promoting regardless of weight. The use of larger groups would be required to elucidate this hypothesis.

In the third study, TRAMP mice fed the high-fat diet as expected gained varied amounts of weight and could be divided into the lightest, obesity-resistant mice having the lightest weights and Obesity-Prone mice the heaviest with the overweight mice between these two groups. However, there were no significant differences among the groups in GUT weights. Further, in this quickly developing model of prostate cancer body weight did not impact age at tumor palpation or at death. The significant differences in tumor weight and volume found in the TRAMP-C2 study was seen while comparing the growth of a very poorly differentiated tumor. However the TRAMP mice fed the high fat diet had tumors that had a variety of differentiation levels. This suggests that a high-fat diet and/or obesity may increase the growth of poorly differentiated aggressive prostate tumors. This is consistent with the results of two meta-analysis of prostate cancer and obesity which concluded that obesity is associated with an increased risk of prostate cancer specific mortality in prospective cohort studies as well as biochemical recurrence [19] and that obesity is associated with a decreased risk of localized prostate cancer but an increased risk of advance prostate cancer [20]. Pathological analysis of GUT revealed obesity-resistant mice had ~70.8% lesions graded as moderately or poorly differentiated adenocarcinoma. Overweight mice had 78.9% moderately or poorly differentiated tumors and there was 91% moderately or poorly differentiated tumor in obesity-prone mice. This finding is consistent with human data suggesting higher body weight is associated with an increased risk of prostate cancer [1]. Taken together these results indicate that overweight and obesity are associated with more severe GUT lesions in the TRAMP mouse.

Neuroendocrine (NE) status as an indicator of poor prognosis has been reported previously in humans [24]. The tumors from TRAMP mice can be either neuroendocrine (NE) positive or negative [51]. To clarify the role of NE differentiation of prostate cancer as it relates to body weight we measured synaptophysin protein expression of the autochthonous tumors. Mice whose tumors were positive for synaptophysin had a significantly younger age of tumor detection as well as an earlier age of death when compared to the NE negative mice. These characteristics were independent of body weight and the amount of fat in the diet suggesting that synaptophysin may represent an independent marker of poor prognosis and that the TRAMP mouse model may be useful in elucidating the mechanisms involved. 

## 5. Conclusion

The role of body weight in prostate cancer can be difficult to differentiate from the role of diet. In addition, prostate cancer is a disease that can take decades to develop making human studies of how diet and body weight impact prostate cancer development extremely difficult. The studies presented here help establish that the TRAMP model can be utilized to examine the effects of weight independently from the effects of diet on prostate tumor progression. In addition, the TRAMP model can be utilized to help determine the role of NE differentiation on the progression of prostate cancer and to establish the potential for diet and weight to influence NE differentiation.

## Figures and Tables

**Table 1:** End-point comparisons for TRAMP GTG male mice^§^.

	Final body weight (g)	Age at tumor palpation (weeks)	Age at death (weeks)	GUT weight (g)	Tumor differentiation			Percent with metastasis
					Well	Moderate	Poor
GTG-6								
Obese (*N* = 4)	51.78 ± 5.30^a^	27.67 ± 4.91 (*N* = 3)	35.75 ± 4.59	6.23 ± 1.40	50%	25%	25%	25%
Lean (*N* = 3)	36.07 ± 3.75^b^	22.0 ± 3.51 (*N* = 3)	28.33 ± 8.84	6.30 ± 1.96	33%	0	67%	67%
PBS (*N* = 15)	36.92 ± 1.33^b^	29.36 ± 1.61 (*n* = 14)	38.33 ± 1.92	7.84 ± 0.84	60%	7%	33%	47%
GTG-26								
Obese (*N* = 8)	48.61 ± 2.60^a^	33.38 ± 0.94	42.13 ± 1.22	9.10 ± 1.54 (*N* = 6)	50%	50%	0	13%
*Lean (*N* = 6)	39.08 ± 2.46^b^	33.50 ± 1.09	43.00 ± 1.67	8.90 ± 1.45 (*N* = 5)	60%	20%	0	20%
PBS (*N* = 12)	36.65 ± 1.63^b^	32.30 ± 1.10	41.30 ± 1.40	8.16 ± 1.0 (*N* = 11)	58%	8%	33%	33%

^§^columns with different letters indicate a significant difference among the groups.

*pathology report not received for one mouse in this group.

**Table 2:** Overview of TRAMP DIO study.

TRAMP DIO	Final body weight (g)	GUT weight (g)	Age at tumor palpation (weeks)	Age at death (weeks)	Metastasis in percent	Tumor differentiation		
						PIN/well	Moderate	Poor
Obesity prone	42.1 ± 1.6 (*n* = 23)	9.4 ± 0.8 (*n* = 23)	29.1 ± 1.2 (*n* = 22)	40.2 ± 2.1 (*n* = 23)	77.3 (*n* = 22)	9.1	45.5	45.5
Overweight	40.0 ± 1.1 (*n* = 23)	8.4 ± 0.8 (*n* = 21)	32.7 ± 1.1 (*n* = 20)	40.8 ± 1.6 (*n* = 23)	57.9 (*n* = 19)	21.1	36.8	42.1
Obesity resistant	36.7 ± 1.3 (*N* = 24)	7.1 ± 0.7 (*n* = 23)	29.4 ± 1.1 (*n* = 21)	36.7 ± 2.2 (*n* = 24)	48.8 (*n* = 24)	29.2	20.8	50.0
Low fat	36.7 ± 1.4 (*N* = 25)	9.0 ± 0.8 (*n* = 21)	30.3 ± 1.1 (*n* = 21)	38.8 ± 2.4 (*n* = 25)	59.1 (*n* = 22)	13.6	50.0	36.4

**Table 3:** Overview of NE status of TRAMP DIO study tumors.

Group	Percent NE positive	NE status	Initial body weight	Body weight increase (6–18 weeks)	Final body weight	Age at tumor detection	Age at death	GUT weight	High grade (%)
Obesity prone	37% (7/19)	Positive	20.8 ± 0.9	14.1 ± 0.6	39.1 ± 2.7	25.1^x^ ± 2.7	30.1^x^ ± 4.3	6.5^x^ ± 1.1	(100)^x^ 7/7
		Negative	20.8 ± 0.7	13.1 ± 0.4	43.9 ± 2.1	31.4 ± 0.9	46.3 ± 0.9	11.4 ± 0.9	(18) 2/11
Overweight	21% (3/14)	Positive	22.8 ± 1.1	9.5 ± 0.4	34.0^x^ ± 2.0	26.7^x^ ± 1.9	29.7^x^ ± 1.9	9.5 ± 0.8	(100) 2/2
		Negative	21.7 ± 0.5	9.5 ± 0.2	41.2 ± 1.2	33.7 ± 1.6	44.6 ± 1.5	9.9 ± 1.0	(56) 5/9
Obesity resistant	46% (6/13)	Positive	21.1 ± 0.5	7.2 ± 0.7	35.0 ± 2.7	26.7 ± 2.6	35.0 ± 4.6	7.7 ± 1.6	(100) 6/6
		Negative	22.3 ± 0.5	8.0 ± 0.2	40.6 ± 3.5	29.3 ± 1.6	42.1 ± 2.9	9.4 ± 10.5	(57) 4/7
All high-fat diet mice	35% (16/46)	Positive	21.3 ± 0.5	10.2 ± 0.8	36.6^x^ ± 1.6	26.0^x^ ± 1.5	31.9^x^ ± 2.5	8.5^x^ ± 0.7	(100)^x^ 15/15
		Negative	21.5 ± 0.3	11.0 ± 0.4	42.1 ± 1.2	31.8 ± 0.8	44.7 ± 0.9	10.4 ± 0.6	(41) 11/27
Low fat	35% (6/17)	Positive	19.7 ± 1.3	8.1 ± 1.8	36.6 ± 2.7	27.2^x^ ± 2.9	35.3^x^ ± 5.0	7.3^x^ ± 1.2	(100)^x^ 6/6
		Negative	21.2 ± 0.6	8.0 ± 0.7	39.7 ± 1.7	31.7 ± 1.2	45.8 ± 1.6	10.4 ± 1.0	(20) 2/10

A significant difference (*P* < 0.05) within each group is designated as ^x^.

**Table 4:** Overview of findings from the three studies.

	Final body weights of GTG-obese mice were similar regardless of age at GTG injection.
	Body and fat pad weights of GTG-obese mice were substantially heavier than GTG-lean or PBS-controls.
TRAMP GTG	GUT weights were not significantly different among the groups.
	Body weight did not significantly influence age of prostate tumor detection or age at death.
	In the 6-week cohort GTG-lean mice tended to have higher rates of poorly differentiated tumors than GTG-lean or PBS-controls.
	Reduced rates of metastases were observed in obese mice.

	At 25 weeks of age body weights of the high-fat fed obesity-resistant mice were lower than the low-fat fed group.
TRAMP-C2 DIO	Tumors from high-fat fed mice were significantly heavier and had significantly larger volumes compared to the low-fat fed mice.
	When body weight was considered the differences in tumor weight and volume were no longer significantly different between any of the groups.
	GUT and prostates from high-fat fed obesity-prone mice were significantly heavier than the other groups.

	Neither body weight nor diet significantly influenced age to tumor detection.
	Tumor differentiation was not significantly different between the groups.
TRAMP DIO	NE status did not differ between groups regardless of body weight or diet.
	NE positive tumors were significantly related to younger age at tumor detection and younger age at death except for in the obesity-resistant groups where there was only a trend.
	All NE positive tumors were poorly differentiated.
